# Stroke Patients’ Characteristics and Clinical Outcomes: A Pre-Post COVID-19 Comparison Study

**DOI:** 10.3390/medicina57050507

**Published:** 2021-05-19

**Authors:** Hong Chuan Loh, Kar Keong Neoh, Angelina Siing Ngi Tang, Chen Joo Chin, Purnima Devi Suppiah, Irene Looi, Khang Wen Goh, Ching Siang Tan, Long Chiau Ming

**Affiliations:** 1 Clinical Research Centre, Hospital Seberang Jaya, Ministry of Health Malaysia, Seberang Jaya 13700, Malaysia; chenjoo_84@yahoo.com (C.J.C.); purnima.crc@gmail.com (P.D.S.); irenelooi@yahoo.com (I.L.); 2 Department of Internal Medicine, Hospital Seberang Jaya, Ministry of Health Malaysia, Seberang Jaya 13700, Malaysia; kkneoh88@gmail.com (K.K.N.); angeltsn88@yahoo.com (A.S.N.T.); 3 Faculty of Computing and Engineering, Quest International University Perak, Ipoh 30250, Malaysia; khangwen.goh@qiup.edu.my; 4 School of Pharmacy, KPJ Healthcare University College, Nilai 71800, Malaysia; tcsiang@kpjuc.edu.my; 5 Pengiran Anak Puteri Rashidah Sa’adatul Bolkiah Institute of Health Sciences, Universiti Brunei Darussalam, Gadong BE1410, Brunei

**Keywords:** coronavirus, ischaemic stroke, thrombolysis, thrombectomy

## Abstract

*Background and Objectives*: The Coronavirus disease 2019 (COVID-19) pandemic caused significant disruption to established medical care systems globally. Thus, this study was aimed to compare the admission and outcome variables such as number of patient and its severity, acute recanalisation therapy given pre-post COVID-19 at a primary stroke centre located in Malaysia. *Methods*: This cross-sectional hospital-based study included adult ischaemic stroke patients. Variables of the study included the number of ischaemic stroke patients, the proportions of recanalisation therapies, stroke severity during admission based on the National Institutes of Health Stroke Scale, functional outcome at discharge based on the modified Rankin Scale, and relevant workflow metrics. We compared the outcome between two six-month periods, namely the pre-COVID-19 period (March 2019 to September 2019) and the COVID-19 period (March 2020 to September 2020). *Results*: There were 131 and 156 patients, respectively, from the pre-COVID-19 period and the COVID-19 period. The median door-to-scan time and the median door-to-reperfusion time were both significantly shorter in the COVID-19 period (24.5 min versus 12.0 min, *p* = 0.047) and (93.5 min versus 60.0 min, *p* = 0.015), respectively. There were also significantly more patients who received intravenous thrombolysis (7.6% versus 17.3%, *p* = 0.015) and mechanical thrombectomy (0.8% versus 6.4%, *p* = 0.013) in the COVID-19 period, respectively. *Conclusions*: The COVID-19 pandemic may not have caused disruptions of acute stroke care in our primary stroke centre. Our data indicated that the number of ischaemic stroke events remained stable, with a significant increase of recanalisation therapies and better in-hospital workflow metrics during the COVID-19 pandemic period. However, we would like to highlight that the burden of COVID-19 cases in the study area was very low. Therefore, the study may not have captured the true burden (and relevant delays in stroke patient management) during the COVID-19 pandemic. The effect of the pandemic crisis is ongoing and both pre-hospital and in-hospital care systems must continue to provide optimal, highly time-dependent stroke care services.

## 1. Introduction

By witnessing the effects of the pandemic and prior to being seriously impacted by COVID-19 itself, the National Stroke Workflow Steering Committee for Malaysia performed a detailed review of the possible collateral effects of the pandemic on stroke patients in Malaysia. The Committee, which consists of neurologists from government (Ministry of Health and university hospitals) and private hospitals, produced guidelines for acute stroke care services during the COVID-19 pandemic. Due to time constraints, the Committee guidelines were introduced instead of the national guidelines for clinical practice since the national guidelines required a more systematic approach and in-depth literature review. Although the Committee guidelines were not as comprehensive, they were very important in such a crisis to assist healthcare workers to minimize the risk of acquiring COVID-19 infection while providing optimal stroke care services [1].

The COVID-19 pandemic greatly affected medical care systems, requiring significant service reorganization in order to ensure continuity of care to patients [2]. Recent studies showed certain negative impacts of the outbreak on acute stroke care resulting in a significant drop in stroke admissions, thrombolysis, and thrombectomy cases [3,4]. However, this does not necessarily mean that the incidence of stroke is declining and the situation could be owing to factors like patients’ fear of contracting the disease while seeking treatment at a hospital, mobility restriction due to lockdown and so forth. Other studies showed there was some evidence that stroke patients delayed seeking treatment during the pandemic [5,6]. This situation, if verified, could cause severe and long-term public health implications. There was also a study indicating that COVID-19 could possibly increase the risk of cardiovascular diseases, including stroke [7]. Therefore, we embarked on this pre-post COVID-19 comparison study to assess the impact of the COVID-19 pandemic on acute stroke care services at a primary stroke centre in Malaysia. Admission and outcome variables such as number of patient and its severity, acute recanalisation therapy, time lapsed to receive treatment were also compared.

## 2. Methods

This cross-sectional hospital-based study was registered with the Malaysian National Medical Research Register and received ethical approval (NMRR-20-1034-55038) from the Medical Research and Ethics Committee, Ministry of Health Malaysia (KKM/NIHSEC/P20-1203 (6) dated 22-July-2020). The Committee waived the need for patient consent.

### 2.1. Setting

Hospital Seberang Jaya (HSJ), as a primary stroke centre, has been providing intravenous thrombolysis to patients in Penang and offering stroke advice to nearby states since 2012. Stroke patients who needed mechanical thrombectomy were referred to a separate comprehensive stroke centre. Even though HSJ was designated as one of the screening centres for COVID-19, the stroke care services were still continued as usual. For stroke patients with an epidemiological link to COVID-19 or with respiratory symptoms, a nasopharyngeal swab would be taken for COVID-19 using the Antigen Rapid Test Kit (RTK-Ag) (SD Biosensor, INC., Suwon-si, Korea). While awaiting the results, these patients would be directed to a quarantine ward. Hospital personnel would equip with full personal protective equipment, i.e., eye protection with face shield or goggles, N95 masks, disposable isolation gowns, long-sleeved plastic aprons, and gloves if they were dealing with potential COVID-19 patients. Stroke patients would receive routine hyperacute stroke care assessment, urgent imaging, and offered revascularization therapy similar to that of the pre-COVID-19 period, regardless of the COVID-19 RTK-Ag result. Further confirmation of COVID-19 with the reverse transcription-polymerase chain reaction test would be carried out after initial stabilization of patients, with an HSJ laboratory turnaround time of around one day. Other precautionary measures to prevent the spread of the disease were introduced, such as restricting all nonessential visits for medical care and replacing them with online phone consultation. The outpatient multidisciplinary stroke clinic was also replaced by the conventional stroke clinic. Nevertheless, the provision of acute stroke care services was not diminished. There was no reduction in stroke beds or other stroke performance measures such as thrombolytic therapy, imaging services, rehabilitation assessment, stroke education, or smoking cessation counselling. Hence, there was no disparity in stroke services or practices between the pre-COVID-19 period and the COVID-19 period in order to ensure timely care of stroke patients during the COVID-19 pandemic.

### 2.2. Data Collection

We included all adult ischaemic stroke patients (Age >18 years old) who presented to HSJ from 1 March 2019 to 30 September 2019 in the pre-COVID-19 period and from 1 March 2020 to 30 September 2020 in the COVID-19 period. Patients’ data were obtained from their medical case records and were verified by a neurologist before data entry and analysis.

### 2.3. Study Outcomes and Statistical Analyses

The variables of the study included the number of ischaemic stroke patients, the proportions of recanalisation therapies among ischaemic stroke patients, stroke severity during admission based on the National Institutes of Health Stroke Scale, functional outcome at discharge based on the modified Rankin Scale, and in-hospital workflow metrics such as onset-to-door time, door-to-scan time, scan-to-reperfusion time, door-to-reperfusion time, and onset-to-reperfusion time. All outcome variables were collected, stratified and compared between the two periods, namely the pre-COVID-19 period and the COVID-19 period.

All data analyses were performed using the Statistical Package of Social Sciences software, version 20.0 (IBM Corporation, New York, NY, USA). Descriptive statistics were employed for all variables in the study. Continuous variables were reported as mean and standard deviation (SD) or as median and interquartile range (IQR) and were compared with the Independent t-test or Mann-Whitney’s U test when the assumptions were not met. Categorical variables were reported as proportions and percentages and were compared with the chi-square test or Fisher’s exact test when the assumptions were not met. All probability values are two-sided and a level of significance of less than 0.05 (*p*-value < 0.05) was considered statistically significant.

## 3. Results

A total of 287 ischaemic stroke patients were included in this study, among them were 131 and 156 patients from the pre-COVID-19 period and the COVID-19 period, respectively. Table 1 presents the characteristics and clinical features of the patients. The mean (SD) age of the patients was 59.6 (13.8) in the pre-COVID-19 period and 61.1 (13.2) in the COVID-19 period. No significant differences in sex, age, and ethnicity were noted between the 2 periods. All risk factors including both the medical-related risk factors (hypertension, diabetes, hyperlipidaemia, or atrial fibrillation) and behavioural-related risk factor (smoking) showed no significant differences between the two periods too. Most of the patients in the two periods presented with mild stroke (median National Institutes of Health Stroke Scale score, 2 versus 3). Patients from the two periods also showed similarities in stroke subtypes, stroke events, and stroke severity. They were discharged with the same mean modified Rankin Scale score of 2.2 and the same median length of stay at the hospital of 2 days. 

**Figure 1:**
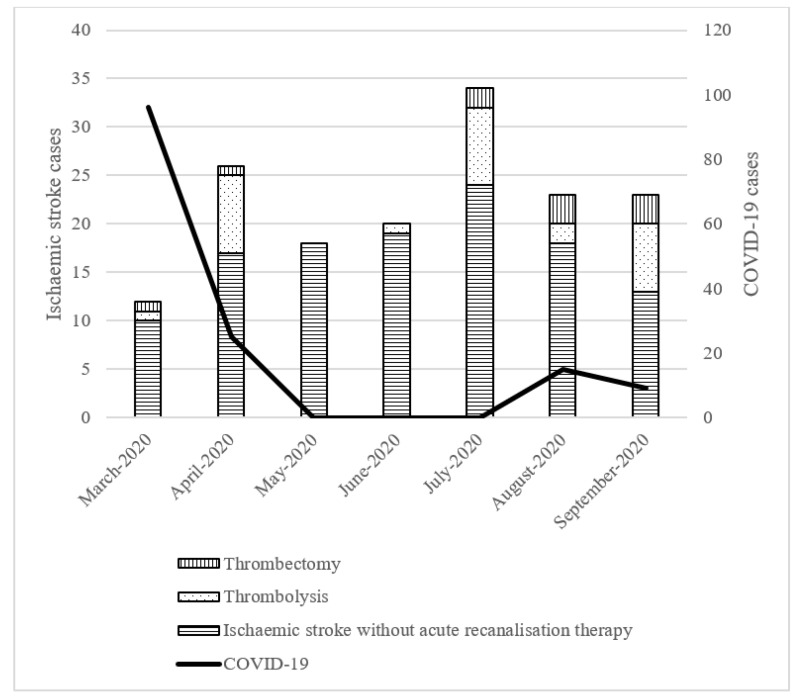
Number of ischaemic stroke cases during the COVID-19 pandemic. Data for COVID-19 positive cases over time in Penang was available for the period beginning in March 2020 and ending in September 2020 (http://covid-19.moh.gov.my/, accessed on 1 May 2021).

The median (IQR) stroke onset-to-door time was 5.6 (9.1) h during the pre-COVID-19 period and 4 (6.9) h during the COVID-19 period. Despite no significant association, the time needed is 1.6 h shorter in the COVID-19 period. The median (IQR) door-to-scan time was significantly shorter in the COVID-19 period, 24.5 (15.0) min versus 12.0 (12.0) min, *p* = 0.047. The median (IQR) door-to-reperfusion time was also significantly shorter in the COVID-19 period, 93.5 (29.0) min versus 60.0 (42.0) min, *p* = 0.015. As depicted in Table 1, there were significantly more patients who received intravenous thrombolysis in the COVID-19 period, 10 (7.6%) versus 27 (17.3%), *p* = 0.015. There were also more patients who received mechanical thrombectomy, 1 (0.8%) versus 10 (6.4%), *p* = 0.013. Figure 1 shows the number of ischaemic stroke cases with or without acute recanalisation and the number of COVID-19 cases in Penang during the COVID-19 pandemic.

## 4. Discussion

Regardless of the additional time necessary for COVID-19 screening on all stroke patients and for the donning and doffing of personal protective equipment by healthcare workers, we noticed that there were no delays in in-hospital workflow metrics compared to the pre-pandemic period. In fact, our study reported significantly shorter door-to-scan time and door-to-reperfusion time during the COVID-19 period. Although our results showed better workflow metrics, which conflicted with previous studies [6,8,9,10,11,12], it was encouraging to observe that, despite unprecedented stress on the medical care system, our primary stroke centre still provided comparable stroke care during the COVID-19 period while strictly abiding by infection control guidelines. These could be due to the fact that with additional training and through reorganization of the medical care system, the readiness of the multidisciplinary team in our centre in response to the pandemic was increased and consequently led to fewer issues in providing stroke care services to patients. Challenges, such as overwhelmed staff in the emergency and intensive care units, inadequate supply of personal protective equipment, and shortages of personnel were thus avoided.

There was a significant increase in the number of recanalisation therapies, in both thrombolysis and thrombectomy cases. One plausible explanation for these findings is better in-hospital workflow metrics, as discussed above. Two other possible explanations were first, large vessel occlusion strokes are often debilitating (i.e., aphasia and/or hemiplegia) and in such a critical situation it is likely that medical personnel would have expedited treatment for the stroke patient despite the pandemic [13]. Second, the health transportation system was at or near full operational strength and was not severely impacted by the COVID-19 pandemic. This would have enabled the smooth transfer of patients who needed mechanical thrombectomy within the therapeutic window to a comprehensive stroke centre. Even though we cannot confirm that the increase in the number of recanalisation therapies was not due to a random fluctuation, we agree with the concept of establishing centralized stroke treatment centres, especially during the crisis, to ensure that there is no disruption of stroke treatment and to prevent the transmission of infectious disease during transportation of patients [14].

We also observed that there was no decline in the number of ischaemic stroke patients between the two periods. This outcome was inconsistent with several other studies that reported a reduction of stroke cases during the pandemic [8,9,11,12]. The severity of COVID-19 cases and the mortality rate in Malaysia were low. The majority (92%) of COVID-19 cases presented with mild symptoms and the mortality rate was only 1.2% [15]. This suggests that the COVID-19 pandemic might not have been seen as a threat and a deterrent by local stroke patients seeking medical help at the hospital. In addition to that, during the pandemic, COVID-19 screening at the emergency department usually took precedence when patients arrived with fever and/or respiratory failure and neurological deficits from a stroke might have been ignored [13]. Based on the result of our study showing stable ischaemic stroke incidences between the two periods, it appears that stroke cases were likely to be recognized by hospital personnel at the emergency department during the COVID-19 period. 

There was no delay in the presentation of acute ischaemic strokes during the COVID-19 period as well. This finding conflicts with another study that reported fewer patients willing to go to hospitals during the pandemic due to the fear of contracting COVID-19 [16]. With the MCO in place, as well as work-from-home allowances from most companies, patients tended to spend more time at home with family, friends and/or relatives. Patients who were not alone at symptom onset demonstrated a higher probability of seeking medical attention [17]. This could be due to the fact that bystanders often recognize stroke symptoms and this situation was positively associated with the decision to call for ambulance assistance [17,18]. Traffic patterns during the pandemic may have also influenced the results of this study. The implementation of restrictions on mobility greatly reduced the amount of traffic. This could be seen in a study in which the overall state-wide traffic volume dropped by nearly half [19]. With less traffic during the pandemic, delays in onset-to-door time were probably less likely to occur. 

Another highlight of our study was the finding that there was no significant difference in stroke severity on admission between the two periods. There was no change in the stroke outcome as well in terms of the modified Rankin Scale score at discharge, number of mortality cases, and the length of hospital stay. These outcomes of the study were consistent with previous studies [10,12]. The COVID-19 lockdown may have triggered behavioural and socio-environmental modifiers that would influence stroke care systems. During the ongoing pandemic, people are more likely to encounter undesired social effects from isolation, such as anxiety caused by fear of falling sick or dying, feeling stranded or abandoned, being stigmatised by others, and from job loss or a failed business venture [20]. A systematic review and meta-analysis demonstrated a substantial increase in the prevalence of stress (29.6%), anxiety (31.9%), and depression (33.7%) during the COVID-19 outbreak [21]. These psychological disorders could be expected to impose more challenges in stroke care. Several studies have reported associations of psychological distress with more severe strokes and a higher prevalence of a poorer outcome [22]. Interestingly, our study suggested that stroke severity and stroke outcome were constant across the two periods despite the possibility that the stroke patients involved may have experienced some degree of psychological dysfunction. However, our study did not include a qualitative approach regarding patient behaviour or a mental health assessment for the patients. Therefore, further study is warranted. 

## 5. Limitation

The following limitations of this study are acknowledged: Firstly, COVID-19 was purportedly related to the increased incidence of cerebrovascular cases [23]. None of the stroke patients included in the study were found to be diagnosed with COVID-19 on admission and no patient developed the disease during follow-up. Studies from China and Korea have found that more than half of COVID-19 patients reported no symptoms at the time of diagnosis [24,25]. Since extensive screening on asymptomatic patients was not carried out in our setting, we simply could not rule out the possibility that our stroke patients had contracted COVID-19 and thus possibly affect the results of the study. Secondly, the full effect of the pandemic is still uncertain and, as the pandemic continues to develop, additional changes may be seen. Our study may not have been of long enough duration to provide clear evidence reflecting the definitive effects of COVID-19 on acute stroke care services. Therefore, continuous monitoring is paramount so that we can observe patterns of the pandemic and adapt our services accordingly. Thirdly, the retrospective nature of our study was limited and was not intended to prove causality or to reaffirm associations and the postulated factors represented a compilation of existing evidence. Fourthly, we would like to highlight that the burden of COVID-19 cases in the study area was very low. Therefore, the study may not have captured the true burden (and relevant delays in stroke patient management) during the COVID-19 pandemic. Lastly, this study represented a dataset from a regional sample in Malaysia and was relatively small. Thus, it might not indicate that there would be a similar response in other societies to the country-specific components of the Malaysian COVID-19 lockdown or to the distinctive stroke care systems in our centre. 

## 6. Conclusions

The study showed no reduction in the number of ischaemic stroke patients but there was an increase in the cases of recanalisation therapy and better in-hospital workflow metrics during the COVID-19 pandemic at our primary stroke centre. The findings of the study, based on preliminary data, provide an insight that might be useful for national policy-makers and could serve to support more studies in geographically diverse regions. Further study is crucial in order to evaluate the emergent care of stroke patients during the rapidly changing landscape of the COVID-19 pandemic so that we can determine the best methods for providing high-quality care while also minimizing the risk of COVID-19 exposure and infection.

## Figures and Tables

**Table 1:** Patient characteristics between the pre-COVID-19 period and the COVID-19 period.

Characteristic	Pre-COVID-19 *n* (%) (*n* = 131)	COVID-19 *n* (%) (*n* = 156)	*p*-Value
Demographics			
Sex, Male	89 (67.9)	98 (62.8)	0.365
Age <65, year	70 (55.6)	90 (59.2)	0.539
Ethnicity			0.414
Malay	69 (52.7)	72 (46.2)	
Chinese	31 (23.7)	51 (32.7)	
Indian	26 (19.8)	28 (17.9)	
Others	5 (3.8)	5 (3.2)	
Risk factors			
Hypertension	94 (71.8)	98 (62.8)	0.109
Diabetes	59 (45.0)	65 (41.7)	0.566
Hyperlipidaemia	21 (16.0)	32 (20.5)	0.330
Atrial fibrillation	3 (2.3)	5 (3.2)	0.731
Smoking	28 (48.3)	36 (34.6)	0.088
Stroke manifestations			
Stroke subtypes			0.681
LACI	95 (77.2)	85 (83.3)	
PACI	20 (16.3)	13 (12.7)	
POCI	5 (4.1)	2 (2.0)	
TACI	3 (2.4)	2 (2.0)	
First stroke event	105 (80.2)	123 (78.8)	0.785
Stroke severity			0.900
None to mild stroke	83 (67.5)	104 (70.7)	
Moderate stroke	37 (30.1)	39 (26.5)	
Moderate to severe stroke	2 (1.6)	3 (2.0)	
Severe stroke	1 (0.8)	1 (0.7)	
Stroke outcome			
mRS at discharge, mean (SD), score	2.2 (1.5)	2.2 (1.6)	0.939
Alive	114 (96.6)	145 (98.6)	0.412
Length of stay <7 days	109 (93.2)	136 (95.1)	0.504
Stroke treatment			
Onset-to-door time <3 h	27 (33.8)	30 (34.1)	0.963
* Door-to-scan time, median (IQR), minute	24.5 (15.0)	12.0 (12.0)	0.047
* Scan-to-reperfusion time, mean (SD), minute	73.3 (31.7)	55.7 (30.2)	0.134
* Door-to-reperfusion time, median (IQR), minute	93.5 (29.0)	60.0 (42.0)	0.015
* Onset-to-reperfusion time, mean (SD), minute	186.7 (45.7)	194.1 (52.3)	0.697
Intravenous thrombolysis	10 (7.6)	27 (17.3)	0.015
Mechanical thrombectomy	1 (0.8)	10 (6.4)	0.013

* Only include ischaemic stroke patients with reperfusion therapies. COVID-19, Coronavirus disease 2019; SD, Standard Deviation; IQR, Interquartile Range; LACI, Lacunar Cerebral Infarct; PACI, Partial Anterior Circulation Infarct; POCI, Posterior Circulation Infarction; TACI, Total Anterior Circulation Infarct; mRS, modified Rankin Scale.

## Data Availability

All data will be available from the corresponding author on reasonable request.

## References

[1] Wan Asyraf W.Z., Ah Khan Y.K., Chung L.W., Kee H.F., Irene L., Ang C.L., Choon W.K., Mak C.S., Tan W.Y., Wn Nafisah W.Y. (2020). Malaysia Stroke Council guide on acute stroke care service during COVID-19 Pandemic. Med. J. Malays..

[2] Baker T., Schell C.O., Petersen D.B., Sawe H., Khalid K., Mndolo S., Rylance J., McAuley D.F., Roy N., Marshall J. (2020). Essential care of critical illness must not be forgotten in the COVID-19 pandemic. Lancet.

[3] Yaghi S., Ishida K., Torres J., Grory B.M., Raz E., Humbert K., Henninger N., Trivedi T., Lillemoe K., Alam S. (2020). SARS-CoV-2 and Stroke in a New York Healthcare System. Stroke.

[4] Zhao J., Li H., Kung D., Fisher M., Shen Y., Liu R. (2020). Impact of the COVID-19 Epidemic on Stroke Care and Potential Solutions. Stroke.

[5] Schirmer C.M., Ringer A.J., Arthur A.S., Binning M.J., Fox W.C., James R.F., Levitt M.R., Tawk R.G., Veznedaroglu E., Walker M. (2020). Delayed presentation of acute ischemic strokes during the COVID-19 crisis. J. Neurointerv. Surg..

[6] Teo K.C., Leung W.C.Y., Wong Y.K., Liu R.K.C., Chan A.H.Y., Choi O.M.Y., Kwok W.M., Leung K.K., Tse M.Y., Cheung R.T.F. (2020). Delays in Stroke Onset to Hospital Arrival Time During COVID-19. Stroke.

[7] Nishiga M., Wang D.W., Han Y., Lewis D.B., Wu J.C. (2020). COVID-19 and cardiovascular disease: From basic mechanisms to clinical perspectives. Nat. Rev. Cardiol..

[8] Paliwal P.R., Tan B.Y.Q., Leow A.S.T., Sibi S., Chor D.W.P., Chin A.X.Y., Yau Y.-W., Cross G.B., Wong L.Y.H., Chia M.L.J. (2020). Impact of the COVID-19 pandemic on hyperacute stroke treatment: Experience from a comprehensive stroke centre in Singapore. J. Thromb Thrombolysis.

[9] Rudilosso S., Laredo C., Vera V., Vargas M., Renú A., Llull L., Obach V., Amaro S., Urra X., Torres F. (2020). Acute Stroke Care Is at Risk in the Era of COVID-19: Experience at a Comprehensive Stroke Center in Barcelona. Stroke.

[10] Kerleroux B., Fabacher T., Bricout N., Moïse M., Testud B., Vingadassalom S., Ifergan H., Janot K., Consoli A., Ben Hassen W. (2020). Mechanical Thrombectomy for Acute Ischemic Stroke Amid the COVID-19 Outbreak: Decreased Activity, and Increased Care Delays. Stroke.

[11] Rinkel L.A., Prick J.C.M., Slot R.E.R., Sombroek N.M.A., Burggraaff J., Groot A.E., Emmer B.J., Roos Y.B.W.E.M., Brouwer M.C., van den Berg-Vos R.M. (2020). Impact of the COVID-19 outbreak on acute stroke care. J. Neurol..

[12] Ghanchi H., Takayanagi A., Savla P., Hariri O.R., Tayag E.C., Schiraldi M., Jorgensen L., Miulli D.E. (2020). Effects of the COVID-19 Pandemic on Stroke Patients. Cureus.

[13] Morelli N., Rota E., Terracciano C., Immovilli P., Spallazzi M., Colombi D., Zaino D., Michieletti E., Guidetti D. (2020). The Baffling Case of Ischemic Stroke Disappearance from the Casualty Department in the COVID-19 Era. Eur. Neurol..

[14] Zhao J., Rudd A., Liu R. (2020). Challenges and Potential Solutions of Stroke Care During the Coronavirus Disease 2019 (COVID-19) Outbreak. Stroke.

[15] Sim B.L.H., Chidambaram S.K., Wong X.C., Pathmanathan M.D., Peariasamy K.M., Hor C.P., Chua H.J., Goh P.P. (2020). Clinical characteristics and risk factors for severe COVID-19 infections in Malaysia: A nationwide observational study. Lancet Reg. Health West. Pac..

[16] Roussel Y., Giraud-Gatineau A., Jimeno M.-T., Rolain J.-M., Zandotti C., Colson P., Raoult D. (2020). SARS-CoV-2: Fear versus data. Int. J. Antimicrob. Agents.

[17] Kothari R., Jauch E., Broderick J., Brott T., Sauerbeck L., Khoury J., Liu T. (1999). Acute stroke: Delays to presentation and emergency department evaluation. Ann. Emerg. Med..

[18] Mosley I., Nicol M., Donnan G., Patrick I., Dewey H. (2007). Stroke Symptoms and the Decision to Call for an Ambulance. Stroke.

[19] Parr S., Wolshon B., Renne J., Murray-Tuite P., Kim K. (2020). Traffic Impacts of the COVID-19 Pandemic: Statewide Analysis of Social Separation and Activity Restriction. Nat. Hazards Rev..

[20] Hall R.C., Hall R.C., Chapman M.J. (2008). The 1995 Kikwit Ebola outbreak: Lessons hospitals and physicians can apply to future viral epidemics. Gen. Hosp. Psychiatry.

[21] Salari N., Hosseinian-Far A., Jalali R., Vaisi-Raygani A., Rasoulpoor S., Mohammadi M., Rasoulpoor S., Khaledi-Paveh B. (2020). Prevalence of stress, anxiety, depression among the general population during the COVID-19 pandemic: A systematic review and meta-analysis. Glob. Health.

[22] Hoyer C., Schmidt H.L., Kranaster L., Alonso A. (2019). Impact of psychiatric comorbidity on the severity, short-term functional outcome, and psychiatric complications after acute stroke. Neuropsychiatr. Dis. Treat..

[23] Merkler A.E., Parikh N.S., Mir S., Gupta A., Kamel H., Lin E., Lantos J., Schenck E.J., Goyal P., Bruce S.S. (2020). Risk of Ischemic Stroke in Patients With Coronavirus Disease 2019 (COVID-19) vs Patients With Influenza. JAMA Neurol..

[24] Day M. (2020). Covid-19: Four fifths of cases are asymptomatic, China figures indicate. BMJ.

[25] Jung C.Y., Park H., Kim D.W., Choi Y.J., Kim S.W., Chang T.I. (2020). Clinical Characteristics of Asymptomatic Patients with COVID-19: A Nationwide Cohort Study in South Korea. Int. J. Infect. Dis..

